# The First Molecular Identification of an Olive Collection Applying Standard Simple Sequence Repeats and Novel Expressed Sequence Tag Markers

**DOI:** 10.3389/fpls.2017.01283

**Published:** 2017-07-19

**Authors:** Soraya Mousavi, Roberto Mariotti, Luca Regni, Luigi Nasini, Marina Bufacchi, Saverio Pandolfi, Luciana Baldoni, Primo Proietti

**Affiliations:** ^1^Consiglio Nazionale delle Ricerche – Institute for Agricultural and Forest Systems in the Mediterranean Perugia, Italy; ^2^Consiglio Nazionale delle Ricerche – Institute of Biosciences and Bioresources Perugia, Italy; ^3^Department of Agricultural, Food and Environmental Sciences, Università degli Studi di Perugia Perugia, Italy

**Keywords:** genetic variability, *ex situ* conservation, germplasm management, genotyping, EST-SSR, *Olea europaea* L.

## Abstract

Germplasm collections of tree crop species represent fundamental tools for conservation of diversity and key steps for its characterization and evaluation. For the olive tree, several collections were created all over the world, but only few of them have been fully characterized and molecularly identified. The olive collection of Perugia University (UNIPG), established in the years’ 60, represents one of the first attempts to gather and safeguard olive diversity, keeping together cultivars from different countries. In the present study, a set of 370 olive trees previously uncharacterized was screened with 10 standard simple sequence repeats (SSRs) and nine new EST-SSR markers, to correctly and thoroughly identify all genotypes, verify their representativeness of the entire cultivated olive variation, and validate the effectiveness of new markers in comparison to standard genotyping tools. The SSR analysis revealed the presence of 59 genotypes, corresponding to 72 well known cultivars, 13 of them resulting exclusively present in this collection. The new EST-SSRs have shown values of diversity parameters quite similar to those of best standard SSRs. When compared to hundreds of Mediterranean cultivars, the UNIPG olive accessions were splitted into the three main populations (East, Center and West Mediterranean), confirming that the collection has a good representativeness of the entire olive variability. Furthermore, Bayesian analysis, performed on the 59 genotypes of the collection by the use of both sets of markers, have demonstrated their splitting into four clusters, with a well balanced membership obtained by EST respect to standard SSRs. The new OLEST (*Olea* expressed sequence tags) SSR markers resulted as effective as the best standard markers. The information obtained from this study represents a high valuable tool for *ex situ* conservation and management of olive genetic resources, useful to build a common database from worldwide olive cultivar collections, also based on recently developed markers.

## Introduction

The cultivated olive (*Olea europaea*, subsp. *europaea*, var. *europaea*, Green, 2002) is one of the most important oil crops in the world and 95% of total olive oil production derives from the Mediterranean basin (Marra et al., 2013; Trujillo et al., 2014). The olive crop counts a very rich varietal heritage, represented by more than 1,200 named cultivars, over 3,000 minor cultivars and an uncertain number of genotypes including pollinators, local ecotypes and centennial trees (El Bakkali et al., 2013; Hosseini-Mazinani et al., 2014; Mazzitelli et al., 2015; Laroussi-Mezghani et al., 2016; Mousavi et al., 2017). Since time of ancient Greece, olive cultivars have been vegetatively propagated, either by cutting or grafting, allowing the accurate reproduction of the best-performing genotypes, leading to the present varietal assortment (Breton et al., 2009; Kaniewski et al., 2012). Thus, most cultivars represent ancient pre-bred genotypes, and the limited and sporadic genetic improvement initiatives, with classical or biotechnological approaches, forced the retention of numerous traditional cultivars despite their agronomical limitations. Among these, only a few have a large area of cultivation and a clear impact on the production of oil and table olives (Ilarioni and Proietti, 2014). But the availability of a large set of well characterized and highly different cultivars is critical to increase the ability to face new agronomical challenges (De Gennaro et al., 2012; Larbi et al., 2015) and future climatic constrains (Moriondo et al., 2013; Proietti et al., 2014; Tanasijevic et al., 2014), diversifying the gene pools, preserving unique genetic traits currently available (Bracci et al., 2011; Corrado et al., 2011; Potts et al., 2012; Klepo et al., 2013) and offering different sensory profiles of extra-virgin olive oils.

Several reasons make it difficult to ensure the identification of cultivars, as the joint cultivation of native and foreign cultivars, the ambiguous plant naming, seedlings or wild plants, or the interchange of plant material over the centuries (Marra et al., 2013; Lazović et al., 2016). Furthermore, the large number of cultivars, the high degree of kinship among many of them, mainly in cases of geographic proximity, and the possible appearance of clonal variation, have raised additional identification problems (Belaj et al., 2007; Caruso et al., 2014; Ipek et al., 2015).

Olive collections represent the main tool to preserve and certify germplasm resources (Belaj et al., 2012; Caruso et al., 2014), mainly when recent trends toward establishing modern orchards exclusively based on a few highly producing and low-vigor cultivars, may potentially lead to the erosion of this germplasm. More than 100 collections of olive genetic resources have been established at international, national and regional levels for conservation and evaluation purposes (Trujillo et al., 2014). A first World Olive Germplasm Bank (WOGB) was established since the years’ 70 at IFAPA (Cordoba, Spain), with about 500 accessions from 21 countries (Belaj et al., 2011; Trujillo et al., 2014). In 2003, a second WOGB was created at INRA (Marrakech, Morocco), including 560 accessions originating from 14 Mediterranean countries (Haouane et al., 2011). An international olive collection built by CNR (ISAFOM) and planted in Zagaria (Enna, Italy), includes about 400 cultivars collected worldwide (Las Casas et al., 2014). A national collection has been built by CREA-OLI (Cosenza, Italy), consisting of approximately 500 cultivars from Italy, corresponding to 85% of total Italian olive germplasm (Muzzalupo et al., 2014). In Turkey, a national olive germplasm collection in Izmir contains 96 genotypes (Kaya et al., 2013), whereas the Greek National Olive Germplasm Collection counts on 47 olive cultivars (Xanthopoulou et al., 2014). Also new olive growing countries, such as the United States of America, have organized important olive collections (NCGR-Davis, CA, United States) (Zelasco et al., 2012), as well as Argentina, Chile, Uruguay, Australia, China, and South Africa (Trujillo et al., 2014). In addition to these important gene banks, many other minor collections were set up along the time to preserve dedicated pools of genotypes, such as cultivars with specific characteristics, wild plants, segregating progenies or core collections (Belaj et al., 2011, 2012; Díez et al., 2011; Marchese et al., 2016). Among these, the UNIPG (Perugia University, Italy) collection, established 50 years ago, represents one of the first attempts to collect and conserve *ex situ* a large number of olive cultivars. It contains genotypes of different geographical origin (although with prevalence of Italian cultivars), and holds great potential for the complete agronomic and exhaustive evaluation of cultivars, as reported by numerous previous works on agronomical, morphological or biological varietal performance (Breton et al., 2014; Portarena et al., 2015).

Simple sequence repeats were the main molecular markers used to characterized the olive germplasm collection (Haouane et al., 2011; Muzzalupo et al., 2014; Trujillo et al., 2014). In fact, SSRs represent the most popular markers for olive genotyping, due to the high polymorphism, extraordinary abundance and fast transferability (Sarri et al., 2006; Baldoni et al., 2009; Díez et al., 2011; Belaj et al., 2012; Hosseini-Mazinani et al., 2014; Mousavi et al., 2014, 2017). However, all SSR loci published so far, characterized by dinucleotide repeat motifs, have demonstrated several drawbacks due to the difficult discrimination among alleles (Baldoni et al., 2009). On the contrary, EST-SSRs derive from expressed regions of the genome, have a greater transferability among species and, since they are located within genes, their variation could find correlation with the phenotype (Duran et al., 2009). However, EST-SSRs may reveal less variations and lower polymorphic information than standard SSRs, eventhough sufficient for population genetic analysis and for genotyping purpose (Yang et al., 2013). For this reason, new trinucleotidic EST-SSR loci recently identified (Mariotti et al., 2016) should now be widely applied for a more clear varietal characterization.

In this work, we have provided the first molecular identification of the accessions present in the UNIPG olive varietal collection. The identification of all olive trees was performed by standard SSRs and, for the first time in olive collections, by EST-SSRs. We intended to reach numerous important goals: (1) the identification of all accessions, including those closely related or morphologically similar, (2) the evaluation of discrimination power between EST-SSRs and dinucleotide standard SSRs, and (3) establishing the level and wideness of the genetic variability inside a germplasm collection, in order to make available this important source of well-defined genotypes to all interested stakeholders and researchers.

## Materials and Methods

### Sample Collection and Archival Records

The Olive Varietal Collection of the University of Perugia – Department of Agricultural, Food and Environmental Sciences (UNIPG) – is located in Prepo, Perugia (43°04′ 53.94″ N – 12°22′ 53.25″ E, altitude about 400 m asl), on a clay soil, with medium content of organic matter, phosphorus and potassium, temperate-Mediterranean climate, average annual temperatures of 12.8°C and annual rainfall of about 900 mm. Planting distance is 5 × 5 m and trees are grown polyconic vase-shaped. Regular agricultural practices are applied to the olive plants, without irrigation. The collection, established in 1965, has been duplicated in 1984 and enlarged by adding further local, national and international cultivars. Based on the UNIPG archive, the collection consisted of 370 olive plants, where each genotype was represented at least by three replications, randomly distributed in a single block, although, some cultivars (Carolea, Maurino, Moraiolo, Leccino, Frantoio, San Felice, Nostrale di Rigali and Manzanilla de Sevilla) were represented by at least 20 trees per cultivar, distributed in four randomized blocks, allowing for their agronomical and morpho-bio-phenological evaluation. No information was available on the original source of plant material.

### DNA Extraction and Molecular Analysis

Leaf samples were collected from each plant, for a total of 370 accessions, and plant position of each tree was recorded. For each accession, total DNA was extracted from fresh leaves following the standard manufacturer’s instructions of GeneElute Plant Genomic DNA Miniprep Kit (Sigma–Aldrich).

All samples were analyzed by using nine best ranked EST-SSR markers (OLEST1-7-9-12-14-16-20-22-23) recently developed (Mariotti et al., 2016). Double step polymerase chain reactions (PCR) were performed in a volume of 25 μl containing 25 ng of DNA, 10× PCR buffer, 200 μM of each dNTP, 10 pmol of primer forward (with 18 bp tail in 5′) and reverse, and 2 U of DNA Polymerase (Q5 High Fidelity DNA Polymerase, New England Biolabs). In the second step, fluorescent tail (10 pmol) was annealed to the forward primer using a double step PCR: the first step consisting in an initial denaturation at 95°C for 5 min, followed by 35 cycles of 95°C for 30 s, 60°C for 30 s and 72°C for 25 s, the second step (for tail annealing) made up of 20 cycles, with the same conditions of the first step except for annealing temperature (Tm = 52°C), a final elongation at 72°C for 40 min closed the second step PCR.

In order to verify the identity of cultivars present in the collection, all samples were genotyped by using standard dinucleotide SSRs markers, widely applied for cultivar characterization in most olive germplasm collections (Haouane et al., 2011; Muzzalupo et al., 2014; Trujillo et al., 2014). Ten high polymorphic markers were applied, including DCA3-5-9-16-18, EMO90, GAPU71B-101-103A and UDO-043 (Sefc et al., 2000; Carriero et al., 2002; Cipriani et al., 2002), previously selected as best performing loci (Baldoni et al., 2009) and common to the other genotyping works. Forward primers carried VIC, FAM, PET, or NED labels at their 5′-end. Standard PCR amplifications were performed in a reaction volume of 25 μl containing 25 ng of DNA, 10× PCR buffer, 200 μM of each dNTP, 10 pmol of each forward and reverse primer, and 2 U of Q5 High-Fidelity DNA Polymerase (New England Biolabs), with an initial denaturation at 95°C for 5 min, followed by 40 cycles of 95°C for 30 s, annealing temperature as suggested by authors (50–60°C) for 30 s and 72°C for 25 s, followed by a final elongation at 72°C for 40 min.

Polymerase chain reactions products were loaded on an ABI 3130 Genetic Analyzer (Applied Biosystems-Hitachi) using the internal GeneScan 500 LIZ Size Standard (Thermo Fisher Scientific). Output data were analyzed by GeneMapper 3.7 (Applied Biosystems).

In order to verify the match of the 370 olive samples with previously characterized cultivars, the data obtained for the 10 standard SSR markers were compared to those available in the database of olive SSR profiles established at CNR-IBBR of Perugia (Italy), including more than 1,000 worldwide olive cultivars, and to other available datasets (Baldoni et al., 2009; Trujillo et al., 2014), allowing to establish cultivar identity and determine all cases of identical profiles, presumably corresponding to clonal genotypes with undetermined presence of mutationsclonal replicates (Baldoni et al., 2009, 2011; Bartolini, 2009; Mousavi et al., 2017).

### Allele Frequency and Diversity Analysis

Number of alleles per locus (*Na*), number of effective alleles (*Ne*), Shannon’s information index (*I*), observed (*Ho*) and expected heterozygosity (*He*), and fixation index (*F*) were calculated at each locus for novel and standard SSRs by the use of GenAlEx 6.501 software (Peakall and Smouse, 2012). Pairwise relatedness was performed on standard and OLEST SSR markers to calculate the allelic similarity for codominant data using GenAlEx 6.501 following the LRM = Lynch and Ritland (1999) estimator – Mean multiplied by 2 to give max of 1.00. The software FreeNA (Chapuis and Estoup, 2007) was applied to detect the presence of possible null alleles (*Fnull*), to determine the genetic uniqueness of each accession and to quantify redundancy. Polymorphic information content (PIC) was calculated for each microsatellite locus using CERVUS v.3.0 software (Marshall et al., 1998). We calculated the probabilities of identity for unrelated individuals [P(ID)] at each locus and across loci, as described by Waits et al. (2001), by using GenAlEx for both OLEST and standard SSR markers. Cumulative P(ID) was calculated by ranking the PIC values at each locus from high to low. We used the criterion of P(ID) lower than 0.001 for the estimation of the minimum number of loci required for individual identification in the study species (Waits et al., 2001).

A model-based Bayesian clustering method was applied to infer the genetic structure of 59 cultivars and to define the number of clusters in the dataset (gene pools) using the software STRUCTURE v.2.3 (Pritchard et al., 2009), for the same sample set separately for OLEST and standard SSRs. Tests were based on an admixture model with independent allele frequencies. No prior information was used to define clusters. Independent runs were done by setting the number of clusters (k) from 1 to 10. Each run comprised a burn-in length of 100,000 followed by 100,000 MCMC (Monte Carlo Markov Chain) replicates. An *ad hoc* statistic ΔK, based on the rate of change in the log probability of data between successive *K* values, as described by Evanno et al. (2005), was calculated through Structure Harvester v.0.9.93 website (Earl, 2012) and used to estimate the most likely number of clusters (k). In order to verify the breakdown of cultivars present in the Perugia collection to the Mediterranean groups previously observed (Sarri et al., 2006), their profiles for ten standard SSRs were analyzed with those of 281 most widely cultivated cultivars of Mediterranean from the CNR-IBBR database by using the same Structure parameters. Data of 281 cultivars were already published (Baldoni et al., 2009, 2011; Mousavi et al., 2017).

## Results

### Polymorphisms Detected at EST and Standard SSR Loci

The nine OLEST markers analyzed were easily scored, showed low stuttering and clear differentiation among alleles (**Table 1**, Supplementary Table S1 and Figure S1). Mean *Na* amounted to 7.9, ranging between 5 (OLEST9) and 15 (OLEST16). *Ne* was 4.466 on average, while the mean *I* value was 1.636. *He* (0.760) was in general higher than *Ho* (0.718), unless for OLEST22 and 23, where *Ho* was significantly higher than *He*. *F* values were positive on average, excluding OLEST22 and 23, and a negligible or moderate amount of null alleles was observed, with no effect on their discrimination power. PIC values were higher than 0.5 at all OLEST loci, with an average value of 0.726 and the maximum discrimination power for OLEST16 (0.848) and OLEST1 (0.804).

**Table 1:** Indices of genetic diversity at 72 cultivars for each SSR locus: number of alleles (*Na*), number of effective alleles (*Ne*), Shannon’s information index (I), observed heterozygosity (*Ho*), expected heterozygosity (*He*), fixation index (*F*) and presence of null alleles (*Fnull*), Polymorphism Information Content (*PIC*).

	*Na*	*Ne*	*I*	*Ho*	*He*	*F*	*Fnull*	*PIC*
**OLEST SSRs**
OLEST1	7	5.750	1.835	0.806	0.826	0.025	0.013	0.804
OLEST7	8	4.056	1.581	0.681	0.753	0.097	0.043	0.715
OLEST9	5	3.660	1.421	0.694	0.727	0.044	0.026	0.681
OLEST12	7	4.056	1.537	0.542	0.753	0.281	0.174	0.713
OLEST14	11	4.852	1.921	0.681	0.794	0.143	0.083	0.775
OLEST16	15	7.210	2.221	0.819	0.861	0.049	0.021	0.848
OLEST20	6	2.742	1.270	0.583	0.635	0.082	0.057	0.591
OLEST22	6	4.213	1.546	0.819	0.763	-0.074	-0.045	0.727
OLEST23	6	3.653	1.390	0.833	0.726	-0.147	-0.068	0.679
Mean	7.9	4.466	1.636	0.718	0.760	0.055	0.034	0.726
**Standard SSRs**
DCA3	11	6.545	2.042	0.889	0.847	-0.049	-0.027	0.830
DCA5	11	2.468	1.364	0.639	0.595	-0.074	-0.076	0.570
DCA9	16	7.551	2.214	0.861	0.868	0.007	0.002	0.853
DCA16	17	6.668	2.266	0.708	0.850	0.167	0.098	0.838
DCA18	13	6.392	2.088	0.903	0.844	-0.070	-0.034	0.826
EMO90	8	3.105	1.420	0.653	0.678	0.037	0.018	0.637
GAPU71B	7	4.324	1.607	0.944	0.769	-0.229	-0.111	0.733
GAPU101	10	6.372	2.019	0.931	0.843	-0.104	-0.049	0.825
GAPU103A	19	9.119	2.454	0.736	0.890	0.173	0.092	0.881
UDO-043	14	6.128	2.099	0.819	0.837	0.021	0.013	0.821
Mean	12.6	5.867	1.957	0.808	0.802	-0.012	-0.007	0.781

**FIGURE 1:**
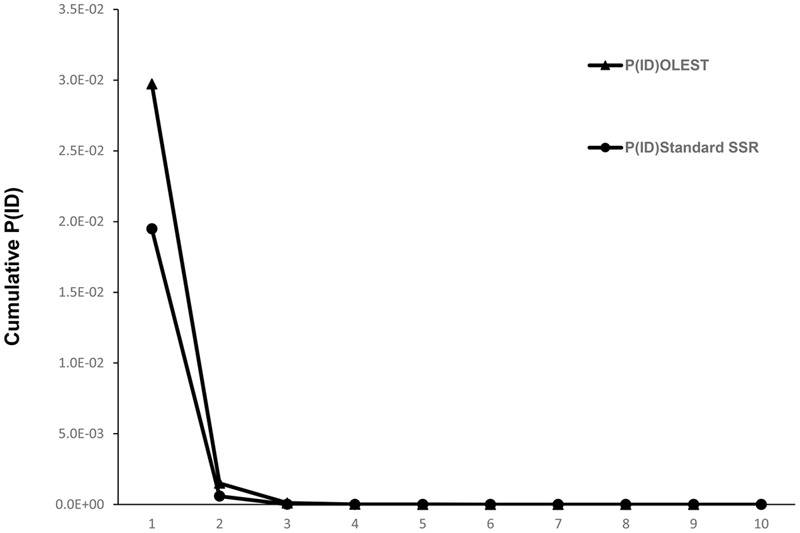
Cumulative probability of identity for OLEST and standard SSRs for unrelated individuals [P(ID)].

Total number of alleles for standard SSRs (**Table 1**) was considerably higher than for OLESTs, with 12.6 alleles per locus. Mean *Ho* was similar to *He* (0.808 and 0.802, respectively), and three out of 10 loci (DCA18, GAPU71B and GAPU101) with *Ho* higher than 0.9. *F* and *Fnull* were slightly negative, showing -0.012 and -0.007, respectively, whereas the mean value of PIC was 0.781. Cumulative probability of identity values (**Figure 1**) showed that a minimum of three loci was required for OLEST markers and only two for standard SSRs to reach P(ID) < 0.001. Therefore, only four and three loci were needed to distinguish all genotypes for OLEST and standard SSR markers, respectively. Nine OLEST [cumulative P(ID) = 2.5e^-10^] or 10 standard SSRs [cumulative P(ID) = 7.3e^-14^] allow for the unequivocal individual identification for this sample set with a high statistical confidence.

### Genetic Identity and Differentiation

The comparison of standard SSR profiles with the CNR-IBBR dataset and previous published data allowed for the identification of UNIPG collection’s samples. Fifty nine distinct genotypes were identified, corresponding to 72 olive cultivars reported in the UNIPG archive. In fact, some samples called in the archive by different names, showed in our work identical genetic profiles (Supplementary Table S1 and **Table 2**). Among genotypes with identical profiles, the first group included the Portuguese cultivars Azeteira and Negrinha, eight cultivars resulted identical to Frantoio (Frantoio Corsini, Razzola, Casaliva, Razza, Taggiasca, Raja Sabina, and Ogliarola di Bitonto) (Group 2), Ogliarola Salentina, Mignola and Cima di Mola formed the third group, Moraiolo, Moraiolo Corsini and Corniolo the fourth, and Dritta di Moscufo and San Felice were the last case of identity. The OLEST markers showed exactly the same results, confirming all cases of identical profiles (Supplementary Table S1).

**Table 2:** Collection code, name of cultivars and Country of origin.

Collection code	Cultivar	Country
PER001	Arbequina	Spain
PER002	Ascolana Tenera	Italy
PER003	Azeteira^G1^	Portugal
PER004	Bella Di Cerignola	Italy
PER005	Canino	Italy
PER006	Carolea	Italy
PER007	Casaliva^G2^	Italy
PER008	Cima Di Mola^G3^	Italy
PER009	Cipressino	Italy
PER010	Coratina	Italy
PER011	Cordovil De Castelo Blanco	Portugal
PER012	Cornicabra	Spain
PER013	Corniolo^G4^	Italy
PER014	Dolce D’andria	Italy
PER015	Dritta Di Loreto	Italy
PER016	Dritta Di Moscufo^G5^	Italy
PER017	Frantoio^G2^	Italy
PER018	Frantoio Corsini^G2^	Italy
PER019	Gentile Di Chieti	Italy
PER020	Giarraffa	Italy
PER021	Gordal Sevillana	Spain
PER022	Grappolo	Italy
PER023	Bella Di Spagna	Italy
PER024	Hojiblanca	Spain
PER025	Itrana	Italy
PER026	Kalamata	Greece
PER027	Konservolia	Greece
PER028	Koroneiki	Greece
PER029	Laurina	Italy
PER030	Leccino	Italy
PER031	Leccio Del Corno	Italy
PER032	Lechin De Sevilla	Spain
PER033	Lucio	Spain
PER034	Madural	Portugal
PER035	Manzanilla De Sevilla	Spain
PER036	Manzanilla Prieta	Spain
PER037	Mastoidis	Greece
PER038	Maurino	Italy
PER039	Mignola^G3^	Italy
PER040	Moraiolo^G4^	Italy
PER041	Moraiolo Corsini^G4^	Italy
PER042	Morellona Di Grecia	Italy
PER043	Moresca	Italy
PER044	Negrera	Italy
PER045	Negrinha^G1^	Portugal
PER046	Nocellara Del Belice	Italy
PER047	Nocellara Messinese	Italy
PER048	Nostrale Di Rigali	Italy
PER049	Ogliarola Di Bitonto^G2^	Italy
PER050	Ogliarola Salentina^G3^	Italy
PER051	Olivago	Italy
PER052	Olivone	Italy
PER053	Orbetana	Italy
PER054	Pasola Di Andria	Italy
PER055	Passalunara	Italy
PER056	Picholine De Languedoc	France
PER057	Picholine Marocaine	Morocco
PER058	Picual	Spain
PER059	Pocciolo	Italy
PER060	Raja Sabina^G2^	Italy
PER061	Razza^G2^	Italy
PER062	Razzola^G2^	Italy
PER063	Rosciola Lazio	Italy
PER064	San Felice^G5^	Italy
PER065	Santa Caterina	Italy
PER066	Santagatese	Italy
PER067	Sourani	Syria
PER068	Taggiasca^G2^	Italy
PER069	Tanche	France
PER070	Tendellone	Italy
PER071	Uovo Di Piccione	Tunisia
PER072	Verdale	France

*G1, G2, G3, G4, and G5: To each group number correspond cultivars showing identical genotype. The underlined cultivars are those exclusively present in the Perugia collection and not in the World Olive Germplasm Banks.*

Eight different countries are represented in the collection, including Italy with 37 cultivars, Spain with nine, Greece with four, Portugal and France with three each, Morocco, Syria, and Tunisia with one each. Thirteen out of the 59 olive genotypes (Dolce d’Andria, Dritta di Loreto, Laurina, Morellona di Grecia, Negrera, Nostrale di Rigali, Olivago, Olivone, Orbetana, Pasola di Andria, Pocciolo, Santagatese, Tendellone) resulted exclusive to this collection and absent in the main WOGBs. Pairwise allelic relatedness performed by GenAlEx showed 100 percent of similarity between the synonymous cultivars (LRM = 1.00) for both set of markers. Comparing OLEST and standard SSRs for allelic similarity the highest values for non-synonymous cultivars were 0.67 and 0.57 respectively, while the minimum LRM values were -0.43 for OLEST and -0.31 for standard SSR markers.

### Population Genetic Structure

From the Structure analysis of data derived from 10 standard SSR loci on the 59 UNIPG cultivars run with 281 Mediterranean representative cultivars (Supplementary Figure S2), the stabilization, in terms of log-likelihood values of ΔK values was observed at *K* = 3 and, assigning individuals to a population for values above 70%, it was observed that 16 cultivars clustered into the Western Mediterranean group, 35 in the Central one and 12 in the Eastern population, only nine genotypes showed high levels of admixture among two or three groups.

**FIGURE 2:**
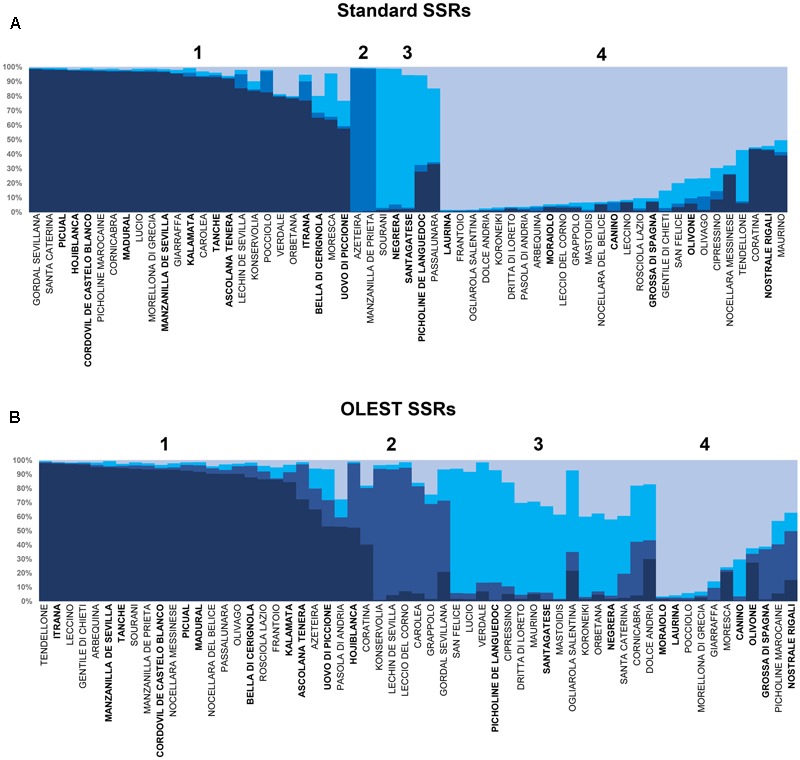
Genetic structure of the 59 cultivars identified at the UNIPG Olive Collection based on data derived from standard **(A)** and new OLEST **(B)** SSR markers. Each vertical bar represents single accessions and colors distinguish the four detected groups. Olive samples with more than one color indicate admixture in their genetic composition. Cultivars assigned to the same group by both kind of markers are reported in bold below every population (from Pop 1 to Pop 4).

The Structure analysis within the cultivars of the collection performed on OLEST and standard SSRs showed the most probable grouping at *K* = 4 (**Figures 2A,B**). Most of the 59 cultivars resulted assigned to two of the four groups for standard SSRs while for OLEST the four structure population were well balanced. In fact, the proportion of membership for OLEST markers was from 0.158 (Pop2) to 0.406 (Pop1), while for standard SSRs the lowest value was 0.054 (Pop2) and for the Pop1 and Pop4 membership value were 0.423 and 0.414 respectively. Only 20 cultivars were assigned to the same population by both set of markers (**Figures 2A,B**). The expected heterozygosity individuated by Bayesian analysis within the same population was on average higher for standard than for OLEST markers (0.84 and 0.76, respectively). Furthermore, the level of population assignment for OLEST markers was lower than standard SSRs (0.75 and 0.88, respectively).

## Discussion

The application of highly effective and discriminant markers may allow the correct identification of all accessions, establishing their representativeness of the species variability and justifying their conservation in *ex situ* collections. This step is crucial to avoid redundancy in germplasm repositories, reducing management costs, distributing true-to-type genotypes for propagation, ratifying reliable genetic sources for breeding programs. The management of germplasm collections, in fact, requires attention and mistakes may be introduced at many stages, from the origin of plant material, that may derive from other collections, private orchards or unreliable sources, to propagation and field planting, and each accession needs correct identification and passport data (Kato et al., 2012; Potts et al., 2012; Trujillo et al., 2014). A thorough and accurate genotype profiling represents a crucial prerequisite to assist breeding programs, perform comparative studies and assess innovative researches.

The collection of olive cultivars established at the University of Perugia represents one of the first efforts to converge into a single set deeply diverse genotypes, deriving from areas with highly different climatic and growing conditions, in order to preserve the variation of cultivated olives and evaluate their characteristics. The genetic identity of genotypes at the UNIPG olive collection was never ascertained before and we were committed to achieve a complete genotyping of all accessions.

Simple sequence repeats markers have become the preferred tool for the identification of olive cultivars, due to their high discrimination power and straightforward data reading (Haouane et al., 2011; Trujillo et al., 2014), however, the largely used dinucleotide SSRs have shown problems related to difficult discrimination between neighboring alleles and low comparability of data among different labs, severely reducing their applicability for large-scale screening (Baldoni et al., 2009) and for comparing the molecular profiles of accessions distributed in different collections (Diez et al., 2015; Torkzaban et al., 2015). For this reason, we decided to apply both, the best ranked dinucleotide SSRs and the recently developed trinucleotide EST-SSRs (OLEST) (Mariotti et al., 2016), in order to also evaluate their reliability in genotyping germplasm repositories.

To establish cultivar identity and determine all clonal replicates, 10 standard dinucleotide SSR markers were preliminarly applied and allele profiles were compared with previously published data (Baldoni et al., 2009, 2011; Hosseini-Mazinani et al., 2014; Trujillo et al., 2014; Mousavi et al., 2017), or included in the CNR-IBBR database. Results derived from these analyses highlighted the presence of 59 distinct genotypes, including five groups of cultivars sharing identical SSR profiles (Bartolini, 2009; Trujillo et al., 2014), but coming from different areas of cultivation and carrying different names.

The same results were obtained when the analysis was independently performed with the new OLEST SSRs: 59 genotypes were distinguished and identified, and the same groups with identical profiles were displayed. Also the values of diversity parameters resulted quite similar to those of best ranked dinucleotide SSRs, particularly for the discrimination power and observed heterozygosity values, with a negligible presence of null alleles. The pairwise relatedness analysis demonstrated the same single-profile groups and highlighted that OLEST markers were more efficient to discriminate among the most polymorphic genotypes, showing the minimum values of allelic similarity.

The occurrence of cases of identical genotype under different cultivar names represents a primary source of problems for identification and a major challenge to the management of germplasm collections (Belaj et al., 2007; Abdessemed et al., 2015). In the olive case (Bradai et al., 2016), as for many other long living trees (Vezzulli et al., 2012; Urrestarazu et al., 2012; Fresnedo-Ramírez et al., 2013; Jiao et al., 2013; Frank and Chitwood, 2016), it can not be theoretically excluded that plant genotypes clonally propagated and living for thousands of years, may accumulate somatic mutations, over the time or as a result of environmental shocks. But these mutations could not be easily revealed by the use of a restricted set of SSR markers and, for this reason, we decided to leave the original names of cultivars, even if they showed the same SSR profile, making them available for future in-depth genomic analyses that would highlight eventual polymorphisms otherwise undetectable (Wu et al., 2014).

By using only three OLEST markers it was possible to discriminate 96.6% of all genotypes. Moreover, OLEST SSRs resulted more easily scorable than dinucleotide SSRs, and didn’t show stuttering problems due to the higher distance among similar alleles and lower slippage during replication. Using the three OLEST markers with the highest PIC values (OLEST1, OLEST14 and OLEST16), 57 out of 59 genotype were discriminated, whereas applying the three most discriminant standard SSRs (DCA09, DCA16 and GAPU103A), all 59 genotypes were completely recognized. In fact, the individual identification estimator [P(ID)] indicates two different accessions may have the same genotype at one specific locus in a population by chance rather than through inheritance, we found that both set of markers were able to clearly distinguish all 59 olive genotypes in the Perugia olive collection.

The Bayesian structure analysis of genotypes present in the Perugia assortment with the wide set of other important cultivars of Mediterranean basin, has shown that the collection well represents the groups in which the cultivated Mediterranean olives were previously splitted (Haouane et al., 2011; Diez et al., 2015), with a higher membership to the Central Mediterranean group, likely due to the prevalence of Italian cultivars. Furthermore, this repository owns 13 cultivars not present in the main international olive germplasm banks (Haouane et al., 2011; Trujillo et al., 2014), strengthening its relevant function for conservation, evaluation and protection of specific genotypes potentially endangered.

When the same analysis was exclusively performed on the UNIPG genotypes, 34% of cultivars resulted assigned to the same population by both sets of markers. The Bayesian results clearly highlighted the differences between OLEST and standard SSRs in the cultivar’s assignment into the structure populations. These dissimilarity was evidenced by the values of expected heterozygosity, the overall proportion of membership and admixture level. Therefore, the results of the present study suggest that, for phylogenetic studies, by using different set of markers could achieve unbalanced assignments. The different ability of both kinds of markers to group cultivars into different clusters could be explained by the nature of OLEST markers as mutations residing in the sequence of transcribed genes, and their alleles could display a higher frequency at regional level, where cultivars were selected based on common characteristics (Biton et al., 2015; Mariotti et al., 2016). Considering that olive domestication process has implied a selection of cultivars for certain agronomic characters, resulting in a loss of genetic variation due to genetic bottlenecks and, in some cases, episodes of founder effect (Cao et al., 2014; Hosseini-Mazinani et al., 2014; Mousavi et al., 2017), EST-SSRs could be related to agronomical traits more than neutral standard SSRs. The very long history of olive growing with several trading events, introduction of alien cultural practices and changes of dietary habits, may have blurred the fingerprints of independent domestication events and led to complex relationships among cultivars (Sarri et al., 2006; Soleri et al., 2010; Díez et al., 2011; Koehmstedt et al., 2011).

The Perugia collection represents the first study case of a real olive germplasm repository validated by standard SSRs and characterized by EST-SSRs. The work has allowed to confirm the OLEST markers as effective genotyping tools, as good as best standard markers for cultivar identification, allowing to avoid the application of other unreliable dinucleotide SSRs. The use of the OLEST markers on a wide set of olive cultivars will help establishing a common fingerprint database without miscalling and binning, exploitable for several molecular investigations, representing a valuable resource for comparative genomics, evolutionary analyses and population studies.

## Author Contributions

SM, LB, LR, PP, MB, RM, LN, and SP contributed substantially to the conception and design of the work; SM, LR, LN, RM, and SP contributed to plant material collection; SM, LB, and RM, performed all molecular work and genotype scoring; SM, LB, LR, RM, LN, and SP interpretation of data; SM, LB, LR, PP, MB, RM, LN, and SP drafted the text; SM, LB, LR, PP, MB, RM, LN, and SP approved the version to be published; SM, LB, LR, PP, MB, RM, LN, and SP agreed to be accountable for all aspects of the work.

## Conflict of Interest Statement

The authors declare that the research was conducted in the absence of any commercial or financial relationships that could be construed as a potential conflict of interest.
