# Supplementary figures and images for: The First Molecular Identification of an Olive Collection Applying Standard Simple Sequence Repeats and Novel Expressed Sequence Tag Markers
Source: Front Plant Sci. 2017 Jul 19;8:1283. doi: 10.3389/fpls.2017.01283 (PMC5515915; doi:10.3389/fpls.2017.01283)

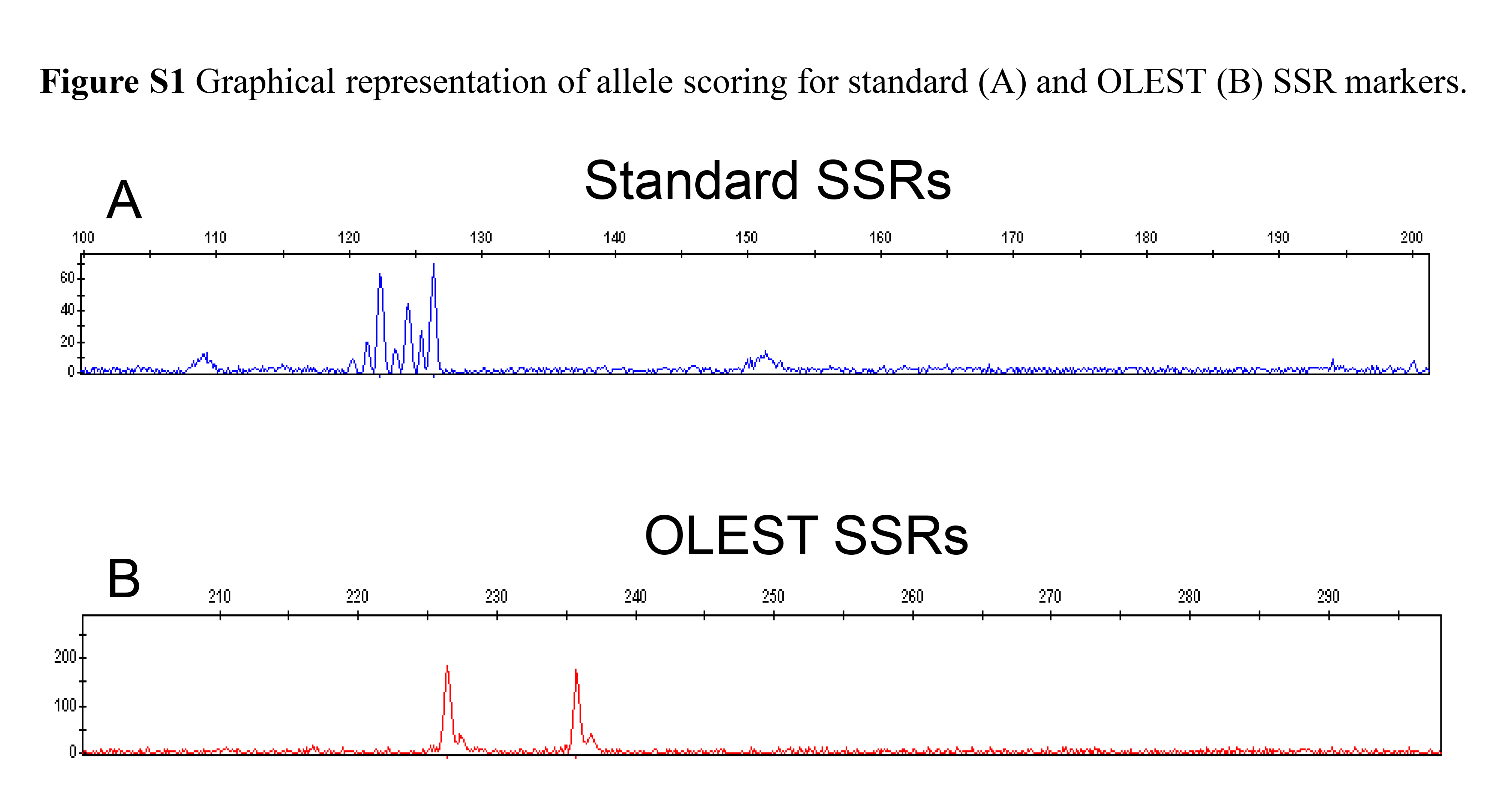

Supplement: Supplementary file 1 [file Image_1.JPEG]
